# Modern Palatant Strategies in Dry and Wet Pet Food: Formulation Technologies, Patent Innovations, and Market Evolution

**DOI:** 10.3390/foods14162824

**Published:** 2025-08-14

**Authors:** Phatthranit Klinmalai, Pitiya Kamonpatana, Janenutch Sodsai, Khwanchat Promhuad, Atcharawan Srisa, Yeyen Laorenza, Attawit Kovitvadhi, Sathita Areerat, Anusorn Seubsai, Nathdanai Harnkarnsujarit

**Affiliations:** 1 Faculty of Agro-Industry, Chiang Mai University, Samut Sakhon 74000, Thailand; phatthranit.k@cmu.ac.th; 2 Department of Food Science and Technology, Faculty of Agro-Industry, Kasetsart University, Bangkok 10900, Thailand; fagipyk@ku.ac.th; 3 Department of Packaging and Materials Technology, Faculty of Agro-Industry, Kasetsart University, Bangkok 10900, Thailand; janenutch.s@ku.ac.th (J.S.); khwanchatpromhuad@gmail.com (K.P.); atcharawan.s@ku.th (A.S.); yeyen.la@ku.th (Y.L.); 4 KU Vet Innova Nutricare Co., Ltd., Kasetsart University, Bangkok 10900, Thailand; attawitthai@gmail.com; 5 Department of Physiology, Faculty of Veterinary Medicine, Kasetsart University, Bangkok 10900, Thailand; sathitameen@gmail.com; 6 Department of Chemical Engineering, Faculty of Engineering, Kasetsart University, Bangkok 10900, Thailand; fengasn@ku.ac.th

**Keywords:** palatability, palatants, pet food, flavor enhancers, encapsulation, canine, feline

## Abstract

Palatability is a critical determinant of pet food performance, directly influencing voluntary intake, nutrient utilization, and therapeutic efficacy. In this systematic review, we examine peer-reviewed research publications, patent filings, and commercial product data pertaining to palatant technologies in dry and wet pet food from 2014 to 2024. Major palatant classes—including fats, proteins, yeast extracts, and novel plant-derived or insect-based hydrolysates—are evaluated for their physicochemical properties, flavor-release mechanisms, and stability during processing. We analyze formulation techniques such as microencapsulation, Maillard-reaction enhancement, and multilayer coating systems, focusing on their impact on aromatic compound retention and palatability consistency. Patent landscape assessment identifies over 15 key innovations in delivery systems, life-stage-specific palatant modulation, and dual-phase release architectures. Dual-phase release architectures are defined as systems that deliver active compounds in two sequential phases, such as immediate and sustained release. Sensory evaluation methodologies—ranging from multivariate preference mapping to descriptive analysis—are critically appraised to correlate human-panel metrics with canine and feline feeding behavior. We also discuss strategic integration of palatants at different processing stages (pre-conditioning, extrusion, and post-extrusion) and the challenges of balancing taste masking with nutritional requirements, particularly in formulations containing alternative proteins for sustainability. Despite rapid market growth in functional palatant-infused products, peer-reviewed literature remains relatively limited, suggesting opportunities for further research on species-specific flavor drivers, synbiotic flavor–nutrient interactions, and novel delivery platforms. This comprehensive overview of palatant science, patent innovations, and market evolution provides evidence-based guidance for researchers, formulators, and veterinarians seeking to optimize organoleptic properties and consumer acceptance of next-generation pet foods.

## 1. Introduction

Palatability plays a pivotal role in the success of pet food products, directly influencing voluntary intake, owner satisfaction, and the overall nutritional efficacy of a diet [1]. While human food consumption is dictated by variety and personal choice, pets rely entirely on the sensory appeal of their food, with aroma, taste, and texture being paramount. To meet this demand, the pet food industry has developed a specialized category of additives known as palatants. These compounds or formulations are designed to enhance the sensory attributes of pet food, ensuring consistent acceptance across species, breeds, life stages, and dietary formats [2].

Traditionally, palatants have included animal fats, protein digests, yeast extracts, and hydrolyzed tissues, often applied as post-extrusion coatings in dry kibble systems [3]. However, increasing pressure for ingredient transparency, clean-label claims, and sustainability has driven significant innovation in palatant development. Emerging approaches now incorporate plant-based proteins, insect-derived hydrolysates, fermented materials, and amino acid–nucleotide systems, often paired with Maillard-reaction technologies to mimic meaty aromas and umami depth [4,5]. These modern palatants are no longer passive flavor enhancers. They are engineered sensory systems designed to work synergistically with base formulations and processing technologies.

Recent patents and commercial innovations have introduced multilayered palatant coatings, emulsified gravies, and life-stage-specific flavor matrices, reflecting a shift from one-size-fits-all solutions to species-tailored palatability systems [6,7]. Moreover, advances in sensory science and animal preference modeling (including two-pan tests, monadic trials, and landscape segmentation analysis) are providing deeper insight into the behavioral drivers of pet food consumption [8].

This review synthesizes recent scientific literature, industrial practices, and patent disclosures from the past decade, with the aim of exploring how palatants are evolving from simple taste enhancers to strategic tools in pet nutrition and product design. Emphasis is placed on ingredient functionality, application technologies, species-specific preferences, and the future directions of palatability science in the global pet food industry.

## 2. Patent and Product Launch Trends in Palatability-Enhanced Pet Foods

**Figure 1:**
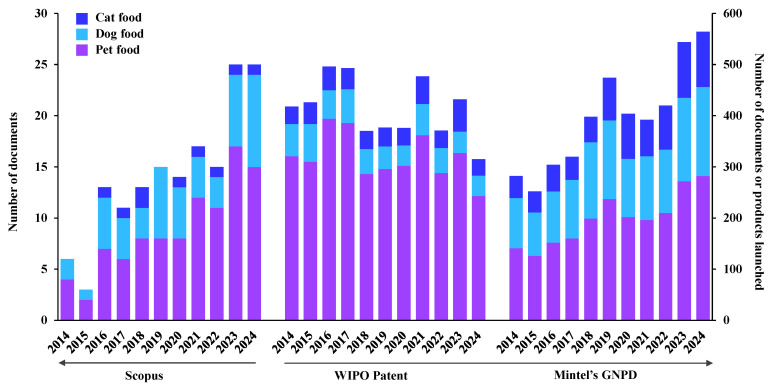
Numbers of documents and products launched based on Scopus, WIPO patents, and Mintel’s GNPD using the following criteria: “pet food”, “dog food”, or “cat food” and the keywords: “palatability” or “palatant” between January 2014 and December 2024.

Palatability is a key factor in assessing the acceptance and preference of pet food. The assessment of sensory attributes and palatability in pet food products commonly involves measuring the intake ratio (IR) and the initial selection between offered diets [9]. Pet food acceptability and preference are traditionally assessed using one-bowl (single-bowl acceptance test) or two-bowl (paired-preference test) methods [10]. Figure 1 shows the number of research publications, patents, and product launches related to pet food palatability from 2014 to 2024. The data indicates that WIPO patents and Mintel’s GNPD record significantly higher numbers of patents and product launches compared to research publications (Scopus). This suggests that industry-driven innovation outpaces academic research, highlighting the pet food sector’s strong investment in developing palatability-enhancing solutions. Furthermore, cat food palatability has gained increasing attention in recent years, due to cats being more selective eaters compared to dogs [11]. Scientific research from 2014 to 2024 indicates that various factors influence the palatability of pet food, including ingredient composition, processing methods, and the role of olfactory compounds in pet food palatability. One prominent trend is the exploration of alternative protein sources to enhance both sustainability and nutritional value. Recent studies have investigated the use of alternative protein sources in pet food, including insect-based proteins [12], macroalgae or microalgae protein [13], keratin from chicken feathers [14], corn-fermented protein [15], and others. These novel ingredients have shown acceptable palatability while contributing to environmental sustainability in pet food. Processing techniques of ingredients or pet food preparation significantly influence the sensory attributes of pet food. For example, high concentration of soybean oil [16], spray-dried animal plasma [17], and corn-fermented protein [18] negatively affected extruder stability and the palatability of dog kibbles.

**Figure 2:**
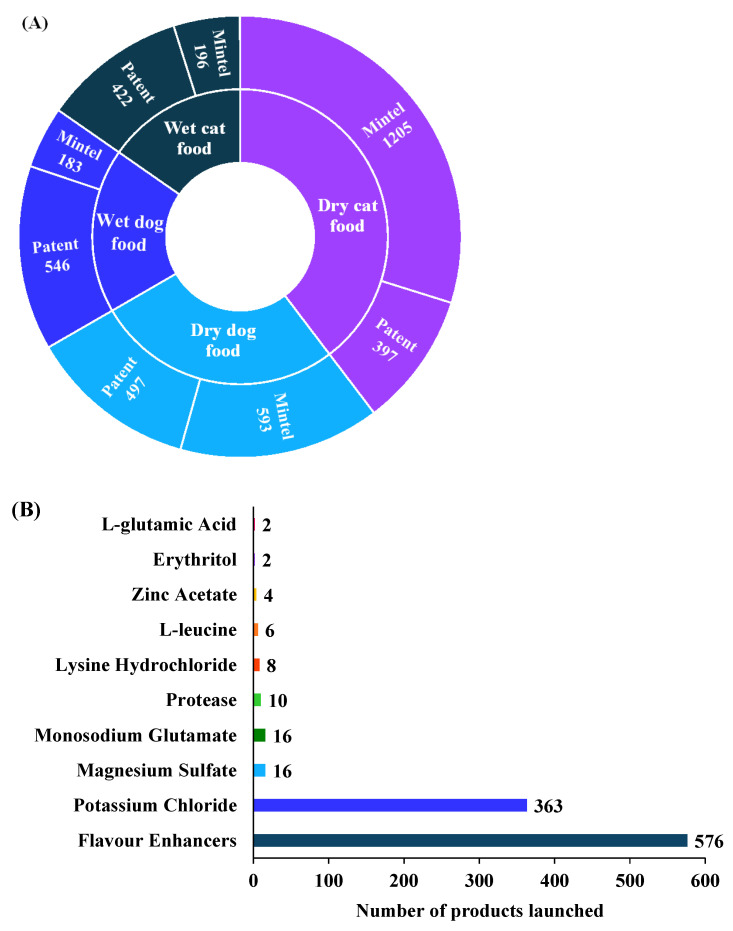
(**A**) Numbers of documents and products launched based on WIPO patents and Mintel’s GNPD using the following criteria: “Wet cat food”, “Wet dog food”, “Dry cat food”, or “Dry dog food” and the keywords: “Palatability” or “Palatant”. (**B**) Top 10 ingredients claimed as flavor enhancers in pet food from Mintel’s Global New Products Database (GNPD) between January 2014 and December 2024.

Figure 2A highlights trends in pet food palatability patent and product launches from 2014 to 2024. Dry cat food leads with 1205 product launches followed by dry dog food (593) that indicates a strong industry focus on dry formulations. Patent applications for dry pet food (397 for cats and 497 for dogs) suggest ongoing innovation in enhancing palatability. Wet pet food shows lower product launches (196 for cats and 183 for dogs) but has higher patents (422 for cats and 546 for dogs) suggesting research efforts despite lower commercialization. This may be due to cost, storage limitations, or consumer preference for dry options. Palatability is influenced by taste, flavor, and texture, which determine the mouthfeel of the food. The addition of flavoring agents can enhance both taste and overall palatability in pet food. Figure 2B highlights the ingredient function used to enhance pet food palatability from 2014 to 2024. Products claimed flavor enhancers are the most frequently used with 576 product launches that indicates their critical role in improving taste acceptance. Potassium chloride follows with 363 launches probably due to its dual function as a palatant and nutritional supplement. Other ingredients such as monosodium glutamate and magnesium sulfate (16 for each) are used in smaller quantities. This suggests a targeted approach to umami enhancement. Protease (10 launches) indicates interest in enzyme-based solutions while amino acids like lysine hydrochloride and L-leucine are incorporated in minimal amounts.

## 3. Scientific Publications on Pet Food Palatability

Palatants are commonly included in pet food to enhance flavor and increase palatability [19]. They can be in liquid or dry form and are coated onto the surface of pet food, such as kibble. Various studies have evaluated the efficacy of different palatants, revealing species-specific responses. A review of palatants used in pet food between 2014 and 2024 is presented in Table 1. Kokemuller et al., (2025) [19] found that the addition of olive extract at 200 ppm was most preferred by cats, while lower concentrations did not significantly differ from the control, and higher concentrations did not negatively affect palatability. Supplementation with an herbal mix containing phosphatidylcholine was preferred by dogs [20]. The secondary metabolites of plants presumably increase food palatability due to the complex odor derived from aldehydes, ketones, esters, sulfur compounds, pyrazines, furans, and alkanes [20]. Alcohols and aldehydes were the dominant volatile compounds in the protein hydrolysate from grass carp waste produced by the Maillard reaction, while hydrocarbons and esters were dominant in commercial attractants [21]. The Maillard reaction’s key pathways diverge from a central intermediate, the Amadori product. This product fragments into dicarbonyl compounds, which then react with free amino acids via Strecker degradation. This crucial step forms volatile Strecker aldehydes, largely responsible for savory, malty, and roasty aromas. Concurrently, other reactive intermediates from the dehydration and cyclization of sugars and amino acids form heterocyclic compounds like furans and pyrazines. Ultimately, these pathways promote the formation of various flavor compounds that contribute to the desirable sensory properties of foods [22]. This self-made attractant increased cats’ acceptability up to 75.37%, compared to the control cat food (37.63%). However, this self-made attractant resulted in lower rates of initial sniffing and first bites by cats than food with a commercial attractant, due to a lower number of volatile compounds. Moreover, the self-made attractant exhibited fewer ketoacid compounds (12 compounds) than commercial attractants (21 compounds); a greater abundance of ketoacids is thought to have a positive effect on palatability enhancement, aligning with cats’ preference for acidic food [21].

Yin et al., (2020) [23] studied the correlation between aroma compounds and the intake ratio/preference test results for six palatability enhancers in dog food. They found nine aroma compounds that were positively associated with the intake ratio: heptanal, nonanal, octanal, (E)-2-hexenal, (E,E)-2,4-decadienal, 2-pentylfuran, 4-methyl-5-thiazoleethanol, 2-furfurylthiol, and (E)-2-decenal. Further testing showed that (E)-2-decenal, 2-furfurylthiol, and 4-methyl-5-thiazoleethanol resulted in higher first choice and consumption ratios. Meanwhile, three compounds (namely (E)-2-octenal, butyrolactone, and methional) were found to be significantly negatively correlated with the intake ratio of dog food.

On the other hand, Wei et al., (2024) [24] also investigated a protein-based food attractant. Studies demonstrate the degree of hydrolysis (DH) in chicken liver hydrolysate plays an important role in cat acceptance, as it results in different levels of free amino acid content and varying peptide molecular mass distribution. These free amino acids and peptides become important flavor precursors in the Maillard reaction to enhance aroma, which can generate key flavor compounds such as aldehydes, ketones, furans, thiophenes, pyrazines, and pyrroles. Feng et al., (2020) [25] reported that dog food with a chicken liver attractant had a higher intake ratio than those with mushroom and insect-based attractants (shiitake mushrooms, *Lentinus edodes*, and yellow mealworm, *Tenebrio molitor*). This finding was related to dogs’ preferences for the meat, roast, fat, caramel, and sour tastes of the chicken liver attractant. The preparation of these attractants by the Maillard reaction results in caramel flavors; combined with sulfur compounds, these can impart a meat flavor to the attractant. Moreover, the heating process, which induces the Maillard reaction, can develop flavor from aromatic compounds and effectively enhance palatability in dog food attractants (DFAs) [26]. They identified 53 aromatic compounds from DFAs through the Maillard reaction, categorized as alcohols, aldehydes, ketones, organic acids, esters, ethers, heterocyclic compounds, phenols, terpenes, and polycyclic aromatic hydrocarbons. However, 23 of these aromatic compounds were correlated with palatability. Specifically, benzaldehyde, vanillin, and 2,5-dimethylpyrazine significantly affected dry dog food preferences. Koppel et al. (2015) [27] found that palatant coatings exhibited a masking effect, reducing the porosity of kibble. Additionally, the palatant coatings eliminated stale, liver, and fish aromas, while strengthening brothy, grainy, and toasted attributes.

Yeast extract exhibits differing palatability effects depending on the food format. In dry kibbles, Oliveira et al., (2016) [28] demonstrated that a blend of 0.5% yeast extract and sodium pyrophosphate significantly improved cats’ acceptance compared to yeast extract alone. The synergy between phosphate salts and yeast-derived umami compounds may enhance aroma release and mouthfeel in low-moisture matrices. In contrast, Lima et al., (2016) [29] found that yeast extract (2%) added to wet cat food failed to improve palatability, with 56.75% of cats preferring the control diet. This discrepancy may stem from matrix-dependent release dynamics; wet food’s higher moisture content could dilute volatile compounds or alter their diffusion profile. Additionally, wet food undergoes different thermal processing (e.g., retort) which may limit Maillard reactions that enhance flavor complexity in dry systems. Thus, the efficacy of yeast extract appears highly context-dependent, influenced by formulation environment, concentration, and potential synergists. In canine studies, Andrade et al., (2019) [30] reported that incorporating 4% spray-dried porcine plasma into extruded pet food reduced palatability, suggesting a negative impact on diet acceptance. Conversely, Polo et al., (2005) [31] found that spray-dried animal plasma (10–20 g/kg) in canned pet food increased protein content, with cats showing a clear preference for plasma over wheat gluten, while dogs exhibited no significant differentiation between formulations. Additionally, Dust et al., (2005) [32] found that processed red blood cells (3%) in extruded kibbles were not preferred by dogs, as 72% opted for the control diet. These findings highlight the complexity of palatant effectiveness, emphasizing the need for species-specific formulations and tailored strategies in pet food development.

Various studies have evaluated the efficacy of different palatants, revealing species-specific responses. Polo et al., (2005) [31] investigated the functional properties of spray-dried animal plasma in canned pet food for both dogs and cats. The inclusion of 20 g/kg of spray-dried animal plasma resulted in a higher protein content compared to 10 g/kg. In palatability tests, dogs showed no significant differentiation between the two products. However, cats exhibited a clear preference for the formula containing plasma over wheat gluten, demonstrating an ability to differentiate and positively select the inclusion of spray-dried animal plasma. Dust et al., (2005) [32] assessed the palatability of extruded kibbles containing processed red blood cells in dogs. The study revealed that dogs preferred the control diet (72%) over the diet containing 3% processed red blood cells (28%). The 3% processed red blood cells diet was not preferred when dogs were offered a choice against a diet containing no processed red blood cells. OliveiraI et al., (2016) [28] investigated the palatability of cat food enhanced with a blend of sodium pyrophosphate and yeast extract. The study revealed that the application of a 0.5% blend (0.2% yeast extract and 0.3% sodium pyrophosphate) to kibbles significantly increased cats’ preference compared to yeast extract alone. This finding suggests a synergistic effect between sodium pyrophosphate and yeast extract in stimulating kibble diets’ palatability for cats. In contrast, Lima et al., (2016) [29] evaluated the efficacy of raw yeast extract as a palatability agent in wet cat food. Studies demonstrate that a 2% supplementation of yeast extract did not improve palatability. Relative consumption results indicated that 56.75% of cats preferred the control diet, while only 33.75% preferred the diet containing yeast extract, and 10% showed ambiguity in their preference. This suggests that yeast extract, when supplemented in its raw form in wet cat food, does not effectively enhance palatability. Andrade et al., (2019) [30] examined the impact of spray-dried porcine plasma on the palatability of dog food. The inclusion of 4% spray-dried porcine plasma in extruded pet food reduced diet palatability, indicating a negative effect on diet acceptance by dogs. Park et al., (2019) [33] explored the effect of fermented medicinal plants as dietary additives on food preference and fecal microbial quality in dogs. The intake ratios of dog food with fermented glasswort and Ganghwa mugwort were significantly higher than that of the control diet (*p* < 0.05). Conversely, the addition of fermented turmeric decreased the acceptance of dog foods. These findings highlight the complexity of palatant effectiveness, emphasizing the need for species-specific formulations and tailored strategies in pet food development.

**Table 1:** Review of palatants used in pet food between 2014 and 2024.

Palatant	Origin of Palatant	Pet	Food Types	Contents	How to Use	Protocol for Palatability Testing	Other Food Tests	Duration of Consumption	Other Major Findings	References
1. Natural olive extract	Olive extract provided by PhenoFarm (Scandriglia RI, Italy)	Cat	Kibble	Liquid palatants: 0, 15, 30, 50, 75, 150 ppm of olive extract Dry palatants: 0, 100, 200, 400, and 600 ppm of olive extract	Coating	Two-day, two-bowl test was conducted with a panel of 20 adult cats. Each cat received an experimental and a control bowl	Olive extract flavor was analyzed by HP-SPME coupled with GC/Q-TOF	2 days	GC-MS analysis identified 27 volatile compounds in olive extract with fruity and earthy flavors Over the 10-day trial, average daily intake was 60–80 g for liquid and 70–80 g for dry palatant, from a 220 g daily serving Cat food palatability was not negatively impacted by adding up to 150 ppm of olive extract to liquid palatant or 600 ppm to dry palatant	[19]
2. Herbal mix: phosphatidylcholine	BioCholine^®^, Nuproxa México -Switzerland	Dog	Kibble	Unsupplemented diet (377 mg choline/kg) was compared with choline chloride (2000 mg/kg) and three levels of a phosphatidylcholine-rich herbal mix (200, 400, and 800 mg/kg)		Comparison between 0 vs. 200 g/kg and 0 vs. 400 mg/kg	Digestibility assay: feces were collectedtwice daily and stored at −20 °C before analysis (dry matter, crude protein, ether extract, ash, and neutral detergent fiber)	30 min for two consecutive days	Dogs consumed a greater quantity of food when the diet was mixed with the herbal blend, resulting in higher intake (0.6) compared to control diet (0.3)	[20]
3. Chicken liver protein hydrolysates	Xinyuan Co., Ltd. (Shanghai, China)	Cat	Neutral cat food from corn flour	Food attractants CFA0 (control), CFA1, CFA2, CFA3, and CFA4 were prepared by spraying pet food with chicken liver protein hydrolysates (CLPHs) of varying degrees of hydrolysis (CLPH1, CLPH2, CLPH3, and CLPH4)	Spraying the cat food attractant into cat food	During the two-bowl test, we recorded the cat’s first choice and the amount of food left in each bowl. We then calculated the intake ratio by dividing the grams consumed by the grams provided	Volatile compound by GC-MS		Cats’ first-choice ratios were: CF4 (70%), CF1 (60%), CF2 (60%), and CF3 (55%) CF4, which contained higher levels of free amino acids and peptides, generated a key flavor during the Maillard reaction	[24]
4. Chicken liver, *Lentinus edodes*, and *Tenebrio molitor*	Chicken and mushroom species	Dog	Dog’s basic food	Control (dog’s basic food), control + chicken liver, control + *L. edodes*, and control + *T. molitor*	Spraying dog food attractant onto cat food	Two-bowl tests recorded ingestion rate (IR) and first preference (FP) as indices of palatability and pet food selection (PFS)	Volatile compound in attractant by HS-SPME-GC-MS and SDE-GC-MS	Two consecutive days, 2 meals per day. The control diet was given within 14 days	Dogs preferred food with chicken liver attractants, showing a significantly higher intake ratio (62.85%) compared to food with *L. edodes* and *T. molitor* (less than 25%). The intake ratio for *L. edodes* was slightly higher than for *T. molitor*	[25]
5. Protein hydrolysate	Grass carp waste attractant prepared by Maillard reaction at pH 7 at 115 °C	Cat	Cat food (blank)	CK: Blank cat food A: Blank cat food + 3% of self-made attractant B: Blank cat food + 3% commercial attractant		Acceptance test: single-bowl feeding test. Palatability test: two-bowl feeding test.	Antioxidant activity (DPPH free radical scavenging ability, hydroxyl radical scavenging ability, Fe^2+^ chelation ability) Volatile compound analysis by GC-MS	Two consecutive days	Acceptability test showed feeding rate values of food with attractants A (75.37%) and B (80.07%) were higher than blank cat food (37.63%) Palatability/preference test showed that the first sniffing and first bite of food attractant A were lower than attractant B, due to lower number of volatile compounds Alcohol and aldehydes were the most common volatile compounds found in the self-made attractant, with concentrations of 34.29% and 33.52%, respectively.	[21]
6. Dog food palatability enhancer powder	Maillard reaction at pH of 6–9 at 100–140 °C	Dog	Basal dog food	Six different palatability enhancers DF1, DF2, DF3, DF4, DF5, and DF6	Spray on basal dog food 1:20	Two-bowl test	Volatile aroma compound (GC-MS, LLME–GC–MS)	Two consecutive days, 2 meals per day	Intake ratio of the six dog food palatants was divided into 3 levels High level: DF1 (82.16%), DF2 (83.45%), and DF3 (80.87%) Medium level: DF4 (40.66%) and DF5 (39.61%) Low level: DF6 (19.08%) Nine aroma compounds were found to be positively associated with intake ratio: heptanal, nonanal, octanal, (E)-2-hexenal, (E,E)-2,4-decadienal, 2-pentylfuran, 4-methyl-5-thiazoleethanol, 2-furfurylthiol, and (E)-2-decenal	[23]
7. Dog food attractant (DFA)	Maillard reaction at pH of 6–9 at 100–140 °C	Dog	Basal dry dog food	Seven DFAs with different aroma compounds (DFA1, DFA2, DFA3, DFA4, DFA5, DFA6, and DFA7)	Spraying seven DFAs onto basal dog food	Eight adult beagle dogs: four males and four females with weight 7.5 to 15.7 kg An acceptance test (one-pan test) and preference tests (two-pan test and free-choice test)	Aroma compounds were analyzed using HS-SPME and GC-MS, and their relationship with palatability was modeled with PLSR	500 g for 2–4 h daily for 5 days	Among 53 aromatic compounds found in DFAs, only 23 aromas were correlated with dog preference and influence palatability Benzaldehyde, vanillin, and 2, 5-dimethyl pyrazines increased the preference for dry dog food	[26]
8. Commercial palatant	D’TECH 6 L obtained from SPF (Descalvado, São Paulo, Brazil)	Dog	Kibble	2% of the ingredients	Coating in a tumble system	Two-pan method with two meals in 1 day for 38 individually kenneled dogs Remains weighed and consumption rate were calculated as relative consumption (%) of each diet	Descriptive sensory analysis: panelists chewed one kibble to evaluate flavor and texture	After a 12 h fast, dogs were offered two pans of experimental food and allowed to eat for 30 min	Brothy, grainy, and toasted flavor increased with reduced oxidized oil and iron aromatics after coating Coating had a masking effect on porosity, stale and bitter taste Fracturability, initial crispness, and grittiness, along with higher dusty/earthy aromatic level, increased the palatability	[27]
9. Sodium pyrophosphate and/or yeast extract	Enzyme extraction from *Saccharomyces cerevisiae*	Cat	Kibbles	0.2% yeast extract, 0.3% sodium pyrophosphate, and 0.5% blends (yeast extract plus sodium pyrophosphate at 40:60)	Faces	Two-bowl method with 20 cats and food relative consumption (%)	-	Two consecutive days and bowls were left with the cats for 24 h	0.5% of the blend (yeast extract and sodium pyrophosphate) increased cats’ preference	[28]
Strain-specific yeast extract	Cellular extraction of the *Saccharomyces cerevisiae* yeast strain cultivated in sugarcane juice	Cat	Wet food	2% of yeast extract	Mixed with wet food	Two-bowl method with 20 adult mixed-breed cats (male and female) and relative consumption (%)	Nutrient digestibility, energy utilization, nitrogen balance, and blood evaluation	Each cat was offered two dietary options once per day, with bowls left for 30 min or until completely consumed	Relative consumption (%) indicated that 56.75% of cats preferred the control diet, 33.75% preferred the diet containing 2% yeast extract, and 10% exhibited ambiguity in palatability preference between the two diets Yeast extract is not a palatability agent in its raw form in wet cat food	[29]
10. Spray-dried porcine plasma (SDP)	Porcine plasma	Dog	Extruded dog food	0, 4, 8, and 12% spray-dried porcine plasma in diet	Added on top of pet food	Pair-wise diet comparison using 20 adult dogs and the intake ratio of the basal diet	Digestibility, facial characteristics, and blood evaluation	30 min for two consecutive days	Diets containing at least 4% SDP increase palatability and digestibility of crude protein and dry matter	[30]
11. Fermented turmeric, glasswort, and Ganghwa mugwort	Turmeric, glasswort, and Ganghwa mugwort	Dog	Pellet	Control, 1% fermented turmeric added to diet, 1% fermented glasswort added to diet, 1% fermented Ganghwa mugwort added to diet, and 1% fermented mixture added to diet	Sprayed uniformly prior to the last oil coating step of extrusion	Two-pan method		Four consecutive days	Intake ratios of the experimental dog food with fermented glasswort and Ganghwa mugwort were significantly higher than that of the control diet (*p* < 0.05). The addition of fermented turmeric decreased the acceptance of dog foods	[33]

**Figure 3:**
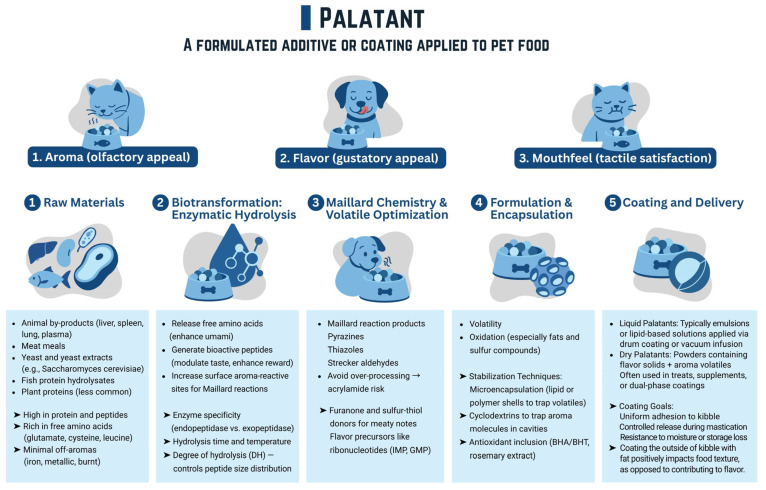
Palatant: A formulated additive or coating applied to pet food.

Formulated additives or coatings applied to pet food represent a sophisticated integration of scientific principles and innovative technologies designed to optimize both palatability (aroma, flavor, mouthfeel) and functionality as shown in Figure 3. The journey of these specialized ingredients begins with the meticulous selection of raw materials, ranging from traditional protein sources to emerging sustainable options like insect proteins or plant-based components, animal by-products, meat meals, yeast, and yeast extracts. This foundational step dictates the potential flavor and nutritional profile, which is typically high in protein and peptides and rich in amino acids. Subsequently, processes like biotransformation, often involving enzymatic hydrolysis or microbial fermentation, are employed to break down these raw materials into more digestible and flavor-rich compounds, such as peptides and amino acids. This biological conversion can unlock unique aroma profiles and enhance bioavailability. Simultaneously, controlled Maillard reactions and volatile optimization are crucial for developing the savory, meaty, and highly attractive notes that are pivotal for pet food acceptance, transforming simple sugars and amino acids into complex flavor molecules through precise heat treatment. To ensure the efficacy and stability of these sensitive ingredients, advanced encapsulation and formulation techniques are indispensable. Encapsulation protects delicate components from degradation during processing and storage, while carefully crafted formulations ensure optimal interaction with the food matrix and controlled release upon consumption [34]. Finally, the coating and delivery methods are critical for uniformly applying these additives to the pet food, ensuring consistent palatability and functionality in every bite. Together, these interlinked stages create high-performance additives that significantly enhance pet food appeal, support health benefits, and meet evolving consumer demands for quality and innovation.

## 4. Palatability Testing

**Figure 4:**
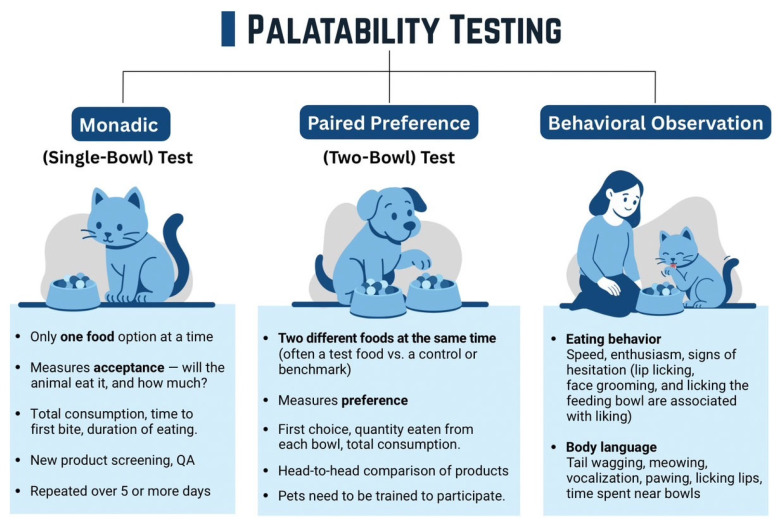
Palatability testing.

Palatability testing is crucial in pet food development as shown in Figure 4, as an unpalatable yet nutritionally complete diet will not be consumed by the animal. Although continuously refined, traditional methods primarily focus on quantifying food intake. The monodic test (or single-bowl test) presents one food type to a pet, measuring the total intake over a set period to determine its overall acceptance. This method is useful for assessing whether a food is consumed at all and often equally fulfills the daily energy requirement. [35]. In contrast, the paired-preference test (or two-bowl test) offers two different food samples simultaneously, allowing pets to choose. Key metrics include first choice (which bowl the pet consume first) and intake ratio (the ratio of amount eaten between two bowls), providing insights into relative preference between diets [35,36]. Pets need to be trained to participate. Beyond intake measurements, behavioral observation offers a complementary, non-consumption-based approach. Researchers meticulously record specific behaviors before, during, and after eating, such as sniffing, lip licking, grooming, and facial expressions, to infer enjoyment or aversion. Body language manifestations include tail wagging, meowing, vocalization, pawing, licking lips, and prolonged periods spent near food bowls. This can provide a more nuanced understanding of a pet’s sensory experience beyond simple food consumption, addressing the challenge that pets cannot verbally communicate their preferences. While traditional tests remain essential, integrating behavioral analysis provides a more holistic assessment of pet food palatability.

## 5. Palatability Influencers

**Figure 5:**
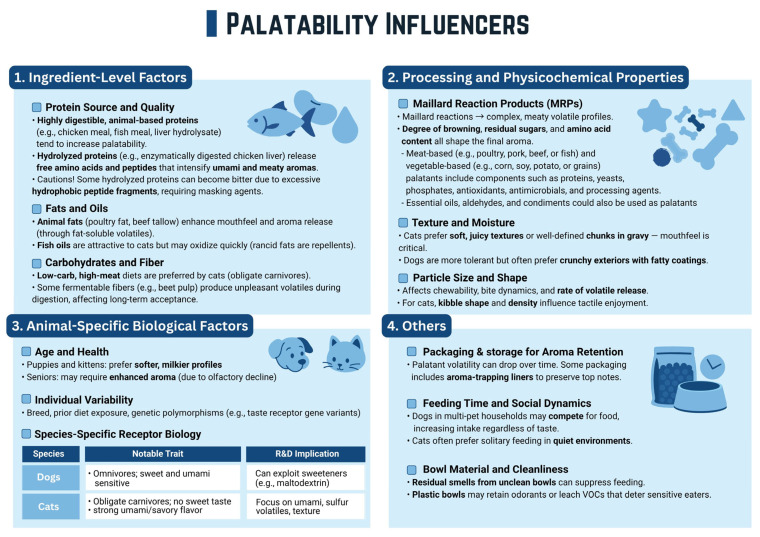
Palatability influencers.

The palatability of pet food is a multifaceted phenomenon, intricately influenced by a complex interplay of sensory attributes, intrinsic pet factors, and the inherent characteristics of the food itself as shown in Figure 5. Understanding these influencers is paramount for formulators aiming to develop highly accepted and consumed pet diets. Firstly, sensory attributes play a pivotal role among ingredient-level factors. Aroma is often the primary driver of initial interest, as pets possess an acute sense of smell, particularly canines. Hydrolyzed proteins release free amino acids and peptides that intensify umami and meaty aromas. Volatile compounds released from the food significantly influence a pet’s decision to approach and investigate a meal. Animal digests, derived from enzymatically or chemically hydrolyzed animal tissues (e.g., poultry meat, pork, beef), are a primary type of palatant. These digests provide rich, meaty, and brothy notes that are highly appealing to carnivorous pets like dogs and cats. Moreover, animal fats enhance mouthfeel and aroma release in pet foods. While fat oils are attractive to cats, they may oxidize quickly. Regarding carbohydrates and fiber, cats prefer low-carbohydrate and high-meat diets. Furthermore, some fermentable fibers, such as beet pulp, produce unpleasant volatiles during digestion, which can negatively affect long-term acceptance. Secondly, concerning processing and physicochemical properties, enzymatic hydrolysis disassembles proteins and fats into smaller peptides, amino acids, and fatty acids. Subsequently, these smaller molecules engage in Maillard reactions during processing, which leads to the generation of flavorful aromatic compounds [9]. Following this, texture becomes crucial, encompassing the food’s shape, size, hardness, crunchiness, and mouthfeel. These physical properties directly impact a pet’s chewing experience and overall satisfaction. Finally, taste, governed by specific chemoreceptors on the tongue, dictates the chemical appeal. While pets respond to traditional tastes like salty, sour, and bitter, their sensitivities vary by species; for instance, cats are renowned for their strong response to amino acids and nucleotides (umami), yet lack a functional sweetness receptor [37,38]. Third, animal-specific biological factors exert a profound influence on palatability [10]. Species differences are fundamental, with cats generally being more discerning and carnivorous, often preferring higher fat and protein levels, while dogs exhibit broader dietary preferences. Breed variations can influence food preferences, often linked to size, jaw structure, or even behavioral traits [39]. Age is another critical element, as puppies and kittens have distinct nutritional and palatability needs compared to adult or senior pets, whose sensory perception and dental health may decline. A pet’s health status also significantly impacts appetite and palatability; illness or medication can alter taste perception and reduce food intake. Lastly, food characteristics are engineered to optimize palatability. The selection and quality of ingredients directly impact the inherent flavor profile, with protein source, fat content, and freshness being key determinants. Processing methods, such as extrusion or baking, profoundly alter texture, release volatile compounds, and induce flavor-generating reactions like the Maillard reaction [40]. Finally, specialized palatants are specifically incorporated as additives to enhance the aroma, taste, and overall appeal of pet food, making it irresistibly attractive to pets [39]. However, other factors also influence food appeal, including packaging and storage conditions for aroma retention, feeding time and social dynamics, and bowl material and cleanliness.

## 6. Current Trends in Palatant Technology

The provided image highlights cutting-edge advancements in pet food palatant technology, showcasing a strategic shift from mere taste enhancement to a more functional approach encompassing gut health, neurological influence, and sustainability. These emerging categories of palatants reflect the pet food industry’s evolving understanding of animal nutrition and well-being.

**Bio-fermented palatants** represent a significant trend towards leveraging natural processes to create unique and appealing aroma profiles. By employing microbial fermentation, manufacturers can transform various substrates into a rich array of volatile compounds, amino acids, and peptides. This biotechnological approach unlocks complex savory and umami notes that are highly attractive to pets, moving beyond traditional thermal processing methods. It also offers a sustainable pathway to enhance the palatability of novel or less conventional protein sources [10].

**Prebiotic-enhanced palatants** combine the primary function of flavor enhancement with crucial gut-health benefits. These innovative formulations incorporate prebiotics, such as mannan oligosaccharides and galactooligosaccharides, alongside conventional palatant carriers like liver digest. The objective is to simultaneously improve food intake and foster a robust, balanced gut microbiome, contributing to better nutrient absorption, improved digestive function, and strengthened immune responses in companion animals [41].

**Targeted neuromodulators** represent a frontier in palatant science. This involves identifying and utilizing compounds that indirectly activate specific neurological pathways, such as the serotonin and dopamine systems, which are associated with reward, satisfaction, and well-being. The aim is to create a deeper, more profound, and lasting positive experience for pets during mealtime, potentially influencing their overall mood and contentment. The primary mechanism involves using specific amino acids or their derivatives, such as tryptophan. As a precursor to the neurotransmitter serotonin, tryptophan can be metabolized by the body into a compound that is key for regulating mood. By including these specific ingredients in a pet’s food, the goal is to enhance the synthesis and availability of serotonin in the brain. This leads to a more profound feeling of contentment and satisfaction, creating a positive experience that lasts beyond the meal itself. Similarly, other compounds may be used to influence the dopamine system, which is centrally involved in the brain’s reward pathways. The combined effect of stimulating both the serotonin and dopamine systems provides a more holistic and lasting positive influence on a pet’s emotional state, turning mealtime into a deeply rewarding experience rather than just a source of basic nutrition [42,43].

**Sustainable palatants.** This trend is driven by environmental concerns and the search for ethically sourced ingredients. It involves the integration of novel protein hydrolysates from alternative sources like insects (e.g., crickets, black soldier fly larvae) or various plant-based peptides [44]. These ingredients are processed to ensure their palatability remains high despite their non-traditional origin, supporting a more circular and environmentally responsible pet food economy. A crucial method for evaluating this shift is through lifecycle assessments (LCAs), which provide a comprehensive, data-driven comparison of the environmental impact of different protein sources from “cradle to grave”. Studies have shown that novel sources, such as insect proteins, often have a significantly lower environmental footprint than traditional meat-based palatants. For instance, the production of insect-based proteins requires substantially less land and water and generates fewer greenhouse gas emissions compared to conventional livestock farming for protein sources like beef or chicken. These novel sources can also be reared on organic waste streams, further reducing their environmental impact and contributing to a circular economy [45]. This critical evaluation through LCAs helps validate the sustainability claims of new palatant sources and guides the industry toward more environmentally responsible practices. The overarching concept of behavioral palatants encapsulates many of these advancements. These palatants are designed to influence a pet’s eating habits and general disposition beyond simple acceptance, often enhanced with pheromone mimics or pheromonal food cues. This could include compounds that promote satiety to aid in weight management, reduce anxiety-related eating behaviors, or simply make mealtime a more calming and positive experience. While often overlapping with neuromodulators, behavioral palatants emphasize the tangible impact on pet well-being.

## 7. Invention Patents of Palatants Used in Pet Food

Table 2 shows the invention patents for palatants used in pet food. The Mars Incorporated. (2016) [6] patent represents a multilayered palatant designed to enhance dry dog food palatability. This technology utilizes a dual plasticizer system, a fat-based outer layer, and structuring agents to achieve a unique textural profile: a crispy exterior, moist visual appearance, and a soft, chewable interior. The addition of acidulants addresses potential off-flavors from glycerin. Animal preference tests demonstrated a substantial increase in consumption compared to standard dry kibble. A novel post-extrusion coating technology [46], detailed in US patent 9585412B2, significantly improves pet food palatability and nutritional profile. This method bypasses thermal degradation by applying a multicomponent coating after extrusion, utilizing controlled fluid dynamics in a mixer. It aligns with the “post-extrusion multilayer coating” technique discussed in Section 3. The technology allows for the incorporation of heat-sensitive and functional ingredients, resulting in increased pet preference and the potential for customized health outcomes. Mikami et al., (2017) [8] detail a methodology for pet food palatability optimization, utilizing human sensory evaluation of individual palatants based on 18 sensory attributes, followed by multivariate statistical analysis to correlate with animal preference data. This combined approach, validated through two-pan tests in dogs, allows for the prediction and formulation of prototype diets with enhanced palatability. Nestlé Purina PetCare Global Resources, Inc. (2024) [47] shows a functional palatant composition for pet food, employing a synergistic combination of fatty acid esters of mono-/diglycerides, glycerol, yeast components, and amino acids. This blend enhances taste and aroma, improves coating stability, and protects aroma-active molecules. The composition’s versatility allows for topical coating or direct blending, enabling tailored palatability for various pet types and food formats. Nestlé Purina PetCare Centre (2019) [7] describes a dual-phase palatability enhancement system for pet food, developed by Nestlé Purina. This method incorporates internal inclusions of palatants (protein digests, fats) during extrusion and applies external flavor coatings post-extrusion. This approach creates distinct sensory zones within the kibble, mimicking moist food textures and flavor release and potentially enhancing consumption. Cayeux, L. (2016) [48] introduces a vitamin-based palatability enhancer composition (PEC) containing vitamin D3 and B1. This composition can be used as a coating or incorporated into pet food and includes animal digests, Maillard precursors, feed carriers (yeast or soy protein), and fats. The combination of these ingredients aims to improve palatability and aroma, especially when subjected to heat treatments that induce Maillard reactions. Sunvold and Corrigan (2016) [49] describe a multilayer coating system for dry pet food, utilizing a protein-rich outer layer, binders (egg white or whey), fats, palatants, and active compounds (probiotics, vitamins). This post-extrusion process, performed in a fluidizing mixer, aims to improve sensory appeal and nutrient delivery. The patent emphasizes controlled application through viscosity and temperature management, catering to growing demands for functional, clean-label, and customized pet food products. Nestec S.A. (2017) [50] introduces a fat-free palatability enhancer for cat food, combining sulfur-containing amino acids (cysteine, methionine) and sugars. Upon thermal processing (80–200 °C), a Maillard reaction generates flavor and aroma compounds. This enhancer, adaptable to various cat food products, improves palatability without adding fat, addressing challenges related to feline taste preferences and specialized dietary needs. This technique can be connected to the “controlled Maillard reaction and volatile optimization” stage (Section 3), demonstrating how sugar–protein systems can be manipulated to generate meaty aromas. It highlights how patented innovations—such as encapsulation, fat-matrix modulation, and life-stage-specific nutrient–palatant integration (Iams Europe B.V., 2020) [51]—directly correspond to raw material selection, biotransformation, and coating/delivery steps in modern palatant formulation. This cross-referencing not only improves narrative coherence but also emphasizes the translational pathway from patented innovation to practical formulation strategies in pet food development. Corrigan, P. J. (2012) [52] outlines a process-oriented approach to enhancing pet food palatability, focusing on the strategic timing and placement of palatant addition during extrusion. This method, including “split dosing”, allows for palatant incorporation at various stages, minimizing volatile compound loss and improving flavor consistency. Emulsifiers and carriers are also used to stabilize palatant dispersion. A Hill’s Pet Nutrition, Inc. (2020) [53] patent presents a method for formulating palatable, structured wet pet food using alginate–calcium gelation to produce retort-stable, meat-like chunks from a protein matrix. Palatability is achieved via a gravy component enriched with animal-derived palatants, ensuring flavor diffusion and retention. This method addresses challenges related to flavor leaching and texture breakdown in wet pet food. Nestlé S.A. (2020) [54] describes a method for enhancing cat food palatability by controlling the ratios of animal and plant fats. Endogenous animal fat (Fa) and endogenous plant fat (Fc) are incorporated during mixing, while exogenous animal fat (Fb) is added during preconditioning, extrusion, or coating. Specific ratios (Fa/Fb: 0.7–2.5, (Fa + Fb)/Fc: 6.0–13.0) are used to create a fat matrix that mimics meat, improving taste preference and aroma delivery. Nestlé S.A. (2017) [55] reports a method for enhancing cat food palatability using a nutritious base (poultry by-products, fish proteins, dried egg, or yeast) and a post-extrusion fat-based coating. The base provides nutritional density, while the coating enhances aroma and flavor. This dual-layer approach, validated through feeding trials, improves acceptance by leveraging cats’ natural preferences and aligning with functional palatant trends. Nestlé S.A. (2014) [5] delineates a palatant system for pet food utilizing a plant-based Maillard reaction. Vegetable protein hydrolysates (wheat gluten), reducing sugars (lactose or glucose), and lipids are combined to generate meat-like aroma and flavor. This system can be incorporated during or after processing, providing flexibility and a clean-label alternative for fat-reduced diets, while maintaining high sensory acceptance. Nestlé Purina PetCare Centre (2017) [56] proposes a method for enhancing wet pet food palatability using meat emulsion systems. Animal muscle tissue, fats, plasma, and optional water-binding agents are emulsified and cooked into retort-stable formats. This allows for controlled texture and flavor delivery, mimicking real meat fibers. Optional palatant-enriched gravy can be added, and the system is adaptable to multiphase wet foods, supporting novel ingredient incorporation while maintaining sensory quality. Iams Europe B.V. (2020) [51] describes a pet food system that adjusts nutrient profiles and palatant concentrations based on life stage. Nutrients like DHA, fiber, and antioxidants are modulated, and palatants are used (in gravies, coatings, or inclusions) to increase acceptance, especially in senior animals. The patent also utilizes packaging with human–animal imagery to improve caregiver compliance, aligning with precision pet nutrition trends. Although patent filings indicate robust innovation in palatant delivery systems, flavor modulation, and functional ingredient integration, a more critical appraisal of commercial viability is warranted. Cross-referencing patent data with actual product launches reveals that only a subset of these inventions achieve market realization. This discrepancy underscores the need to evaluate not just technological novelty but also regulatory feasibility, cost-effectiveness, scalability, and consumer acceptance—key factors influencing the translation of patented formulations into commercially successful pet food products.

## 8. Challenges and Future Opportunities

A key challenge lies in maintaining palatant efficacy and stability throughout complex manufacturing processes and extended shelf lives, especially for novel bioactive components. A significant hurdle, particularly for new ingredients like insect or plant-based proteins, is navigating the complex regulatory landscape, such as achieving Generally Recognized as Safe (GRAS) status in the US. This process can be both costly and time-consuming, often requiring extensive scientific data and safety evaluations that can take months or even years to complete. Furthermore, the stability of palatants is a critical concern, especially under extreme storage conditions like those found in tropical climates. High heat and humidity can accelerate the degradation of flavor compounds, leading to a loss of aroma and taste intensity. For example, research has shown that palatants, if not properly stabilized with antioxidants or other preservation systems, can lose a significant portion of their potency over time, resulting in a product that becomes unpalatable to pets long before its expiration date. Understanding the nuanced and diverse sensory preferences across different pet species, breeds, and life stages remains a complex endeavor, requiring more sophisticated and standardized evaluation methodologies. Furthermore, the sustainability and ethical sourcing of traditional animal-derived palatants present growing concerns, pushing the industry towards alternatives. However, these challenges pave the way for substantial opportunities. The development of bio-fermented palatants offers a sustainable route to unique, highly palatable aroma profiles. Prebiotic-enhanced palatants represent a dual benefit, combining taste appeal with gut health advantages. Future innovations will likely focus on targeted neuromodulators, compounds that subtly influence pet behavior and well-being through gut–brain axis interactions. The integration of insect protein hydrolysates and plant-based peptides will address sustainability concerns, while advanced analytical techniques and predictive modeling will revolutionize palatant discovery and formulation. Ultimately, the industry is moving towards highly customized, functional palatants that not only entice pets but also contribute holistically to their health and satisfaction. Moreover, while current evaluation methods—such as one-bowl (monadic) and two-bowl (paired-preference) tests—are widely used, their lack of methodological uniformity across studies and manufacturers limits cross-comparison [1,10]. A consensus-based framework is needed to integrate intake metrics, behavioral observation, and volatile compound profiling, enabling consistent interpretation across canine and feline studies. Furthermore, by combining the chemical fingerprinting of palatants with historical preference data, machine learning algorithms could predict acceptance across species, life stages, and dietary formats. This would reduce the reliance on iterative trial-and-error feeding tests. For example, such models could integrate data from GC–MS volatile profiles [23,24] with animal preference datasets [8] to forecast palatability outcomes. This approach aligns with emerging trends in predictive formulation and precision pet nutrition.

## 9. Conclusions

Palatants have evolved into a cornerstone of modern pet food formulation, bridging the gap between nutritional adequacy and sensory appeal. As companion animal owners become increasingly discerning, the demand for highly palatable, functional, and species-appropriate diets continues to rise. This has driven innovation not only in the selection of palatant ingredients—ranging from animal digests to novel protein hydrolysates, yeast derivatives, and amino acid–nucleotide blends—but also in application technologies, such as multiphase coatings, emulsified gravies, and Maillard-enhanced systems. Scientific and industrial advancements have made it possible to design palatants with greater flavor complexity, improved aroma retention, and targeted appeal to specific breeds, life stages, or dietary needs. Furthermore, sensory testing protocols and behavioral assessment tools have refined how palatability is measured and optimized, ensuring product success in increasingly competitive markets. Despite these advancements, challenges remain. The rise of plant-based and alternative protein diets, for example, introduces taste-masking obstacles and shifts in consumer perception that must be addressed through innovative palatant strategies. Likewise, regulatory, sustainability, and nutritional constraints require palatants to perform beyond flavor alone—supporting not only intake stimulation but also digestive tolerance, shelf life stability, and clean-label compliance. In conclusion, the future of palatants in pet food lies in multifunctional systems that integrate ingredient science, processing control, and sensory behavior modeling to deliver holistic pet food solutions. As the field advances, palatants will continue to shape the palatability, performance, and perception of pet nutrition on a global scale.

## Figures and Tables

**Table 2:** Invention patents of palatants used in pet food.

Types of Palatant	Origin of Palatant	Pet Type	Food Types	How to Use	Major Claim	Country of Patent Applicants	Patent Name	References
1. Fat		Dogs	Dry pet food	Applied as external coating (post-extrusion), inner inclusion in granule	Improved palatability, softness, and texture through multilayered coating	Russia (RU)	Attractive fodder for domestic animals and methods for improving attractiveness of fodder for domestic animals	Mars Incorporated. (2016) [6]
2. Protein	Egg	Dogs and Cats	Dry pet food	Applied post-extrusion using fluidizing mixer	Enhanced palatability and nutritional value via protein–palatant–fat coating system with reduced thermal degradation	United States	Process for making a pet food in the form of a coated kibble	Corrigan (2017) [46]
3. Protein		Dogs	Dry pet food	Palatants were sprayed on dry base food (DS-A) at 0.2% w/w for testing; preference measured via 2-pan test	Innovative method for evaluating pet food preference using mapped sensory analysis and preference data to optimize formulation without requiring humans to consume pet food	Japan	Method for evaluating food preference of pets	Mikami et al. (2017) [8]
4. Fatty acid Yeast Amino acid		Dogs and Cats	Dry pet food	Applied as topical coating Blended into food formulation	Improved palatability and acceptance by combining fatty acid esters, amino acids, and yeast-based flavor systems	International	Methods and Compositions for Palatable Pet Foods	Nestlé Purina PetCare Global Resources Inc. (2024) [47]
5. Protein digests Fats		Dogs and Cats	Dry pet food	Internal inclusions added before extrusion External coating post-extrusion Layering optimized using multistep application system	Enhanced sensory experience and palatability through spatially distinct placement of palatants (inside and outside the kibble); mimics meaty textures and flavors	Canada	Methods and Systems for Making Food	Nestlé Purina PetCare Centre (2019) [7]
6. Vitamin D3 Vitamin B1 Fat	Maillard precursors	Dogs and Cats	Dry, semi-moist, and wet pet foods	Added by coating or inclusion (before processing); optional heat-treatment to improve flavor development (e.g., Maillard reaction)	Improved palatability through vitamin-based enhancers in combination with carriers and flavor precursors; adaptable to various food forms and species	South Korea (KR)	Palatability Enhancers for Pet Food, Method of Preparation and Uses Thereof	Cayeux, L. (2016) [48]
7. Protein Fat Probiotics Vitamins	Egg white Whey	Dogs and Cats	Dry pet food	Post-extrusion coating in fluidizing mixer	Enhanced palatability, nutrient delivery, and stability through sequential, multilayer coating with functional and flavor-enhancing components	Australia	Pet Food in the Form of a Coated Kibble	Sunvold and Corrigan (2016) [49]
8. Amino acid		Cat	Dry, semi-moist, and wet cat foods	Applied as coating or internal inclusion; optionally heat-treated (80–200 °C for 10 s to 210 min) to generate Maillard-derived aroma and flavor compounds	Improved palatability in cat food using a fat-free system based on amino-carbonyl Maillard reaction products; adaptable to various moisture levels and food formats	Japan	Preference Improver Containing Amino Reactant and Carbonyl Compound for Use in Cat Food	Nestec S.A. (2017) [50]
9. Fat		Dogs and Cats	Dry pet food	Palatants added at various process stages Post-extrusion coating	Improved and consistent palatability by timing palatant addition at key production points; reduced loss of aroma compounds and improved consumer acceptance	International	Process for Making Pet Food	Corrigan, P. J. (2012) [52]
10. Meat analogues formed	Alginate–calcium gelation	Dogs and Cats	Wet pet food	Forms meat-like structures pre-retort Mixed with gravy containing palatants	Preparation of retort-stable, striated, restructured meat analogues using alginate–calcium gelation, suitable for canned/gravy-style pet foods	United States	Process for Preparing a Pet Food Composition	Hill’s Pet Nutrition, Inc. (2020) [53]
11. Fat	Animal fats Plant fats	Cats	Dry pet food	Fat incorporated internally and coating post-extrusion	Enhanced palatability by controlling specific fat ratios from animal and plant sources; formulation shown to outperform standard kibbles	Japan	Specific Fat Fraction Containing Palatable Cat Kibble	Nestlé S.A. (2020) [54]
12. Protein Fat	Poultry by-products Fish materials, dried whole egg, yeast	Cats	Dry pet food	Palatants included in formulation and also applied post-extrusion as coating	Enhanced palatability through optimized nutritional composition and targeted coating with attractant palatability agents	Japan	Taste Dry Cat Food and Method for Producing the Same	Nestlé S.A. (2017) [55]
13. Protein hydrolysates Reducing sugars Fat	Wheat gluten Lactose Glucose	Dogs and Cats	Dry and wet pet foods	Mixture incorporated during production or applied post-processing	Improved palatability by generating desirable Maillard reaction products from plant-based protein, sugar, and lipid systems	International	Processed Food Such as Petfood with Improved Palatability	Nestlé S.A. (2014) [5]
14. Emulsified meat-based matrix	Ground animal muscle Fat Plasma	Dogs and Cats	Wet pet foods	Meat emulsion prepared and cooked into stable chunks or paste and mixed with gravy containing palatants	Improved palatability, texture, and water retention in wet pet foods via emulsified meat matrices	Canada	Meat Emulsion Products, Methods of Making Such Products and Pet Foods Containing Such Products	Nestlé Purina PetCare Centre (2017) [56]
15. Palatant concentration		Dogs and Cats	Dry and wet pet foods	Palatants integrated into each life-stage-specific formula or adjusted in level to drive voluntary intake	Life-stage-specific animal diets optimized in nutrient profile and optionally palatant concentration, enhancing selection and compliance through human–animal life stage visual cues	Netherlands	Compositions and Methods for Providing a Life-Stage Appropriate Animal Diet	Iams Europe B.V. (2020) [51]

## Data Availability

The data presented in this study is available on request from the corresponding author.
